# New Zealand *Pae Ora* Healthcare Reforms 2022: Viable by Design? A Qualitative Study Using the Viable System Model

**DOI:** 10.34172/ijhpm.2023.7906

**Published:** 2023-12-06

**Authors:** Adeel Akmal, Nataliya Podgorodnichenko, Robin Gauld, Tim Stokes

**Affiliations:** ^1^Department of General Practice and Rural Health, Dunedin School of Medicine, University of Otago, Dunedin, New Zealand; ^2^Department of Business Studies, University of Iceland, Reykjavik, Iceland; ^3^DBA Programme, Otago Business School, University of Otago, Dunedin, New Zealand; ^4^Department of Management, Otago Business School, University of Otago, Dunedin, New Zealand

**Keywords:** Health Reforms, Viable System Model, Health Policy, Qualitative Research

## Abstract

**Background:** The New Zealand (NZ) *Pae Ora* (Healthy Futures) health reforms came into effect in July 2022 with the establishment of Health New Zealand (HNZ) (Te Whatu Ora) and the Māori Health Authority (MHA) (Te Aka Whai Ora) – the organisations charged for healthcare provision and delivery. Given these changes represent major health system reform, we aimed to conduct an early evaluation of the design of the reforms to determine if they can deliver a viable and sustainable NZ health system going forward.

**Methods:** The evaluation was informed by Beer’s viable system model (VSM). A qualitative exploratory design with semi-structured interviews and documents analysis using thematic analysis was used. We conducted 28 interviews with senior healthcare managers and reviewed over 300 official documents and news analyses.

**Results:** The VSM posits that for a system to be viable, all its five sub-systems (operations; co-ordination; operational control; development and governance) need to be strong. Our analysis suggests that the health reforms, despite their strengths, do not satisfy this requirement. The reforms do appreciate the complexity of the healthcare environment: multiple stakeholders, social inequalities, interdependencies. However, our analysis suggests a severe lack of detail regarding the implementation and operationalisation of the reforms. Furthermore, resourcing and coordination within the reformed system is also unclear.

**Conclusion:** The health system reforms may not lead to a viable future NZ health system. Poor communication of the reform implementation and operationalisation will likely result in system failure and inhibit the ability of frontline health organisations to deliver care.

## Background

**Key Messages**

**Implications for policy makers**

- Early evaluation of health system reforms is possible using the viable system model (VSM).
- Detailed information about the implementation and operationalization of the 2022 New Zealand (NZ) health reforms is missing.
- There is a lack of attention given to the future development and sustainability of the health system in the reforms.
- The “wicked problems” of health systems must be acknowledged in health system reforms and dealt with accordingly.

**Implications for the public**

This study adds evidence that the New Zealand (NZ) health reforms 2022, despite their aspirations to improve healthcare quality and equity, do not satisfy the requirements for a future viable health system. There is a significant lack of information about the implementation of changes and what those changes will entail for the healthcare sector and the many stakeholders who help in the health provision and delivery. There is much existing research evidence that shows a lack of operational and implementational information inhibits success in such endeavours. Approaches to balance centralisation and local autonomy, which are at the heart of these reforms, need to be developed to make these reforms viable. It is possible that the reformed system might create additional coordination problems by simultaneous increase in both centralisation and local autonomy. If such challenges are not addressed, the reforms’ implementation will be seriously jeopardised.

In Aotearoa New Zealand (NZ), following on from a comprehensive review of the current health system in 2020,^1^ the government announced system-wide health reforms in 2021 to address the challenges faced by the health system.^2^ On July 1, 2022 the reforms — known as the *Pae Ora* (Healthy Futures) reforms — took effect. From early 2020 to July 2022 there were a series of announcements made by the government at conferences and in official documents which outlined the key features of the reformed system. The majority of these announcements were made available on a specially curated website (futureofhealth.govt.nz) of the Department of Prime Minister and Cabinet.^2^

 The shortcomings of the previous NZ health system and its inability to deliver equitable health outcomes for Māori and Pacific peoples are well recognised.^3^ The previous system has also been criticised for a lack of consistency and coherence in healthcare delivery, insufficient engagement with consumers and problems around access to care, particularly for rural populations.^4^ As a result, four key objectives for the healthcare reforms were announced: equitable health outcomes; consistency and coherence in healthcare delivery; people-centred care, and equitable access.^2^

 At a structural level, the reforms disestablish the 20 district health boards (DHBs) who were tasked with funding and delivering health services at local level. Instead, Health New Zealand (HNZ) (Te Whatu Ora) and the Māori Health Authority (MHA) (Te Aka Whai Ora) take over this responsibility.^5^ These national entities, along with the Public Health Agency (PHA) and the Ministry of Health (MoH), form a national health system. In theory, this restructure should streamline administrative and operational functions and enable more rapid development and implementation of health policy.^6,7^

 Such national reforms, however, create turbulence in the affected sector and more widely,^8^ requiring major resources and commitment from multiple stakeholders.^9,10^ They also engender high hopes and aspirations in society.^11,12^ It is therefore imperative that health system reforms achieve desirable outcomes and develop viable system—characterised by long-term sustainability. This will not be straightforward.^13-15^

 Healthcare problems represent problems with high levels of structural, generative, dynamic, communicative, and social complexity (See Box 1).^16-19^ The viability and success of health system reforms are predicated upon their ability to manage complexity both exogenous (environmental) and endogenous (associated with internal structures and activities).^20-22^ As the NZ health reforms are at an early stage of implementation, it is timely to evaluate whether they are *designed *to build a viable and sustainable health system. The viable system model (VSM) can be used as a framework for such evaluation.^23,24^ We therefore aimed to assess the design of the 2022 NZ *Pae Ora* health reforms using the VSM.

**Box 1. Complexity Features of Healthcare: A Wicked Problem**

It is almost impossible to disentangle multiple factors that influence human health and well-being.^29^ This includes genetics, physical environment, living conditions, education, access to healthcare facilities and medication, level of health knowledge development and many other factors.^30-33^

There is no shared definition of good health and well-being. These concepts are largely culturally dependent.^34-36^ However, these definitions direct the search for solutions and the development of healthcare approaches and systems. This highlights an ultimate impossibility to develop a single best solution to suit everyone.

Health systems involves multiple stakeholder groups with different perspectives, values, needs, cultural norms, and interests.^37^ Among these groups are communities characterised by many factors such as age, gender, ethnicity, culture, different socio-economic status, geographical location and various health status; healthcare providers; governments; research institutions; and different types of healthcare delivery professionals. For example, in NZ, the health needs are different for Māori and non-Māori communities,^38^ for those who live in cities and in rural areas,^39,40^ and for communities with differing socio-economic status.^32^ The healthcare system needs to address this complexity balancing multiple, often clashing values, accommodating tensions and ensuring that proposed solutions address inequities.^41-43^

Public health is a relentless problem which does not have any permanent solutions and quest for better health and improved well-being will never stop.^44-46^

------------------

Abbreviation: NZ, New Zealand.

**Figure 1:**
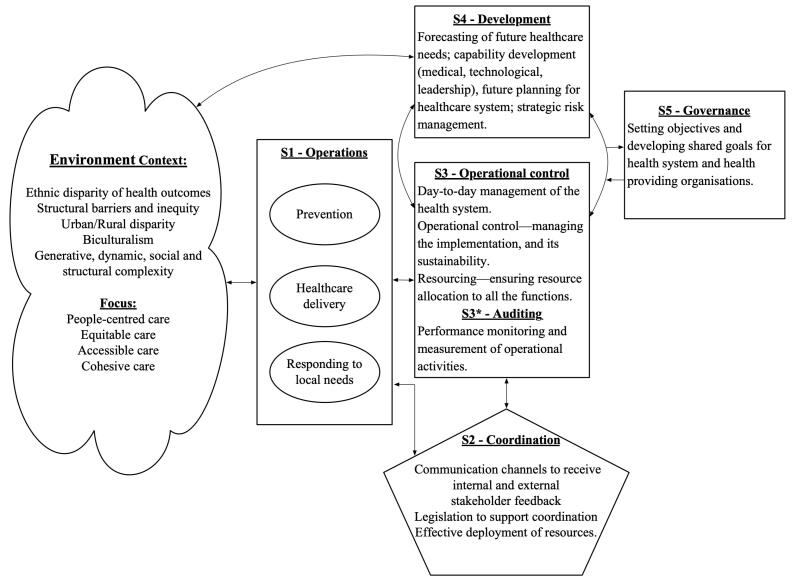
Viable System Model of a Healthcare System.

VSM was developed to identify the key principles which undergird *viability* of any system: its ability to endure changes and turbulence associated with the complexity and variability, and sustain independent existence.^25,26^ VSM can be used as a problem structuring methodology which helps to organise and communicate information about the complexity of a social organisation.^27^ VSM suggests that in any system, five sub-systems (System 1 to 5) are a necessary condition for viability (See Table 1).^28^ These sub-systems include value creation (System 1), coordination (System 2), operational control and auditing (System 3 and 3*), development (System 4), and governance (System 5). Systems 1 and 4 are outward looking — they ensure that the system can match and absorb environmental complexity. Systems 2, 3, and 5 deal with internal variety and complexity and ensure the cohesion of the whole system.^22^ Also, the principles of recursiveness and communication channels are key to system’s functioning. The concept of recursion suggests that systems embedded within a larger system contain all the same elements and have the same structure discussed above. Communication channels create connections between the system and its environment as well as within the system and among its hierarchical layers, and among sub-systems 1-5. Figure 1 illustrates.

**Table 1:** Viable System Model

**System **	**Definition**
System 1 – Operations	System 1 implements an organisation’s purpose by interacting with organisational environment and delivering its primary activities to achieve the ultimate objectives of the system. To deliver its key activities, the system needs to be structured/organised to be able to match the complexity of the environment and tasks it has to perform.^22^ Therefore, in our analysis of System 1, we analysed how the activities of the NZ health reforms are structured and how this structure of activities absorbs health system’s complexity identified above while achieving the reforms’ objectives.
System 2 – Coordination	System 2 focuses on coordination among the parts and units of System 1 ensuring diminishing tensions, smooth communication as well as efficient and effective deployment of resources by all parts of an organisation.^26^ System 1 and 2 are closely interrelated, as attempts to match the environmental complexity may often create structures which put pressure on coordination.^28^ To enable coordination of complex-structured systems such mechanisms as policies and legislation,^24^ scheduling systems,^23^ standardisation, shared ethos, and common language and narratives are developed.^22^
System 3 – Operational Control and Auditing	System 3 is responsible for the internal and immediate functions of the enterprise: its “here-and-now,” day-to-day management.^25^ System 3 deals with operational control, resourcing, and performance measurement and management. In this regard, System 3 is supported by System 3* — auditing, which informs performance measurement. Overall, the role of System 3 and 3* is to ensure that the plans set for the whole system are implemented.
System 4 – Development	System 4 focuses on the development and future orientation of a system ensuring that the System is informed of future developments and threats in its environment. It works closely with System 3 which based on its performance measurement system informs the System 4 of disruptions and emergencies as well as any new knowledge about changes that may enable or hinder progress. In countrywide reforms, System 4 may be focused on constant learning and developing dynamic capabilities which will enable the system to proactively deal with arising challenges.
System 5 – Governance	System 5 is concerned with the governance: setting objectives, developing a shared identity, managing the system and implementing policy. It provides a clear focus for the system — purpose and ethos. In a health system, System 5 would be focused on the communication and integration of the system objectives to the operations of the various entities operating on the system’s frontline providing health services to the population.

Abbreviation: NZ, New Zealand.

## Methods

###  Study Settings

 To evaluate the viability of the system, the evaluation must be conducted with an understanding of the NZ context. NZ has a diverse population comprising of indigenous Māori people, Pacific peoples, European New Zealanders and recent immigrants. There are both urban areas and dispersed rural populations. Due to historical trends and geographical location, the diverse populations of NZ have significantly different health needs and experience inequitable health outcomes.^47^ The publicly funded health system was designed to deliver health and well-being outcomes for the whole population. In 2001, 21 DHBs (later reduced to 20) were established for funding and provision of health services in their geographical regions.^48^ In 2002, primary health organisations (PHOs) were established under Primary Healthcare Strategy as non-profit organisations with community and provider representation. They were funded via the DHBs on a capitation basis and considered a key component of the country’s primary healthcare system. PHOs are responsible for delivering a range of essential health services to their enrolled populations.^49^

 To meet the needs of diverse populations, the 2022 *Pae Ora* health reforms introduced localities — geographical networks that focus on meeting the health and well-being needs of the local communities, giving stronger voice to iwi, mana whenua, hāpori Māori and other community stakeholder groups.^50^

 The research process to explore and evaluate the development and design of the *Pa Ora* reforms began in September 2021 by following the news and updates from the then Transition Unit (assigned to design and deliver the reforms by the Department of Prime Minister’s Cabinet). As DHBs were to be abolished and replaced by HNZ, and the reforms prioritised community health and well-being, it was deemed significant to connect with the PHOs instead of the DHBs for interviews and data collection. PHOs were being debriefed by the Transition Unit on the reforms, it was clear that their roles and responsibilities will be revised after the reforms, and they will be assisting with selecting the prototype locality networks, that were announced in April 2022.

###  Study Design

 This study uses an interpretive qualitative approach.^51,52^ It is informed by VSM as an analytical framework.^28^ Primary (interviews with healthcare managers) and secondary data (official documents as well as the news articles and analyses about the reforms) were collected. It is part of a larger study exploring whether NZ locality network partnerships improve health system performance and outcomes.^53^

 Healthcare managers from four large PHOs, and the Transition Unit (a government unit assigned to assist and develop the reforms’ policy and oversee the transition to the new system) were interviewed. PHOs were chosen because unlike the DHBs, PHOs are still functioning in the reformed system, and their employees as well as the registered general practices with each respective PHO will be directly affected by the changes inherent in the reforms. It is suggested that they may transform into network support agencies for the proposed locality networks — health networks based on local needs of the population. PHOs under the previous health system formed part of local alliances providing district-wide healthcare governance and furthering integrated care.^54^ The work of these alliances will inform the new planned locality networks. Interviewing PHO staff helped to understand the frontline changes to health provision and delivery as well as the implications of the overall reforms for the general public. Moreover, members of the Transition Unit and HNZ, especially those who were involved with localities development, were interviewed too. Using a purposive sampling method,^55^ senior managers and policy analysts were recruited and interviewed.

 Official documentation from the NZ government and the MoH was also collected. The documentation was primarily retrieved from the health reforms website *futureofhealth.govt.nz* which communicated the official information about the upcoming reforms. A total of 99 documents were retrieved. Furthermore, 237 news articles and analyses from major NZ newspapers and healthcare-related magazines such as *NZ Doctor* were also retrieved (See Supplementary file 1).^56^

 All documents containing details or the discussions of the *Pae Ora* health reforms were chosen for the analysis. While the information from *futureofhealth.govt.nz* mainly described the objectives, aspirations, procedures, and future agenda related to the implementation of the reforms, the newspaper articles provided perspectives of various stakeholders and highlighted the challenges in the health system that the health reforms should tackle.

###  Data Collection and Analysis

 Semi-structured interviews using Zoom^®^ were conducted by AA between November 2021 to May 2022. The interview schedule was developed by AA and TS and refined after subsequent interviews by all the research team (See Supplementary file 2 for the interview schedule). Interview questions focused on the perceived aims and objectives of the reforms; operationalization of the reforms especially the locality networks; the enablers and challenges of implementing the reforms objectives; and the perceived outcomes of the reforms over the long term.

 All interviews were recorded, transcribed verbatim and analysed using a thematic analysis approach,^57^ and using Nvivo^®^ to improve reliability in data analysis.^58^ As well as the interviews, informal group discussions with the same participants were conducted throughout the data collection period. These discussions were used to help develop the key themes as preliminary findings were provided back to the participants for their input and clarification.^59^

 The official documents and the news articles were also coded with the same approach. The key themes from the document and interview analyses were compared, and later integrated using the VSM framework. Hence, the primary data from the interviews and the secondary data from collected documents complemented each other and strengthened the rigor of the analysis.^60^ Our goal was to seek further appreciation in the form of expert opinions and analyses as well as a criticism of the reforms, and their operationalization.^56^ The news articles served that purpose.

 The first round of coding was conducted by two researchers (AA and NP), and shared with the other team members (TS and RG) to ensure a consensus on coding. We used a “stepwise-deductive inductive” approach in the thematic analysis.^61,62^ This allowed us to combine inductive generalisations with deductive specialisations for each of the top-level themes related to the health reforms and their development. Initially, data were coded inductively. The VSM framework was then used to aggregate the individual codes into themes. Hence, researchers went back and forth between the data and the theoretical lens of VSM to make sense of the major themes with respect to the VSM.^28^ Research rigor was supported by prolonged engagement with the participants through informal discussions^59,63^; data triangulation^64^; and member checks^59,65^; use of an interview schedule and Nvivo^®^ software for coding.^58^ Further, the consolidated criteria for reporting qualitative research were followed to ensure the trustworthiness of the research.^66^ It is presented as a supplementary file 3 in this paper. Furthermore, research rigor was supported by prolonged engagement with the participants through informal discussions^59,63^; triangulation of primary and secondary data^64^; and member checks^59,65^; use of an interview schedule, Nvivo^®^ software for coding^58^ and, finally, two researchers developed a coding scheme and coded the data with the rest of the team checking the coding accuracy.

## Results

 Twenty-eight interviews and 14 informal group discussions (with 22 out of the 28 participants) were conducted. The interviewees’ demographic characteristics are presented in Table 2. Using the VSM as the basis of the analysis, we explore the overall viability of the NZ health reforms 2022 and provide an overview of each of the VSM sub-systems 1 to 5. Illustrative participant (P) and document (D) quotes are presented.

**Table 2:** Participants’ Demographic Information

**Characteristics**	**Frequency**
Gender	
Male	12
Female	16
Positions	
Senior manager	10
Middle manager	12
Frontline employee	6
Organisations	
PHO	19
Transition unit	9
Ethnicity	
Māori	7
Pacific peoples	5
Pakeha and others	16

Abbreviation: PHO, primary health organisation.

###  System 1 – The Structure of Value Creation

####  Strengths 

**Figure 2:**
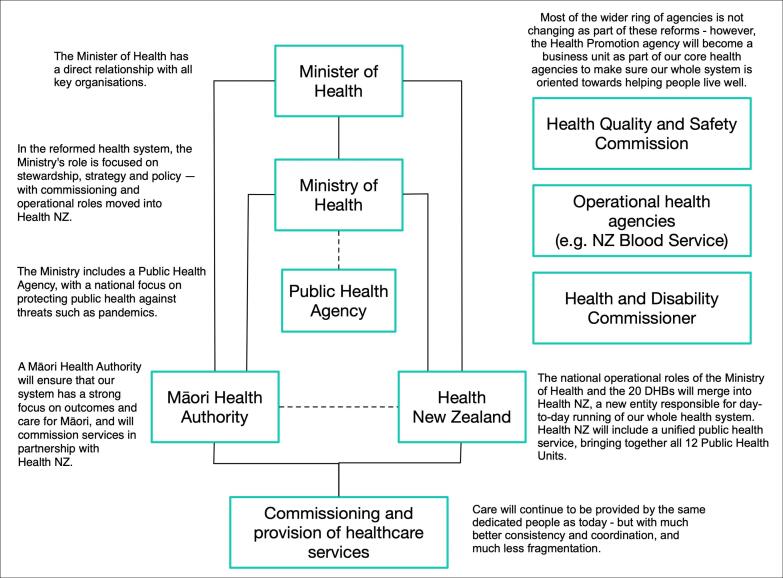
Structure of the Reformed Health System.Abbreviations: NZ, New Zealand; DHBs, District Health Boards.

 The design of system 1 demonstrates several strengths: it addresses environmental complexity and contributes to the achievement of key objectives of the reforms through the development of a new system structure (See Figure 2, and for the old health system structure, please see Supplementary file 3). The newly formed locality networks serve to address complexity associated with the geographical landscape of NZ (multiple regions, urban centres, and remote rural areas). Also, the customer complexity (diversity of population) is addressed via the formation of the MHA as well as the localities which will partner with local community and population group representative organisations such as the local Iwi and Rūnanga (Māori). The MHA serves to ensure that Māori needs, interests, values, and cultural norms are identified and prioritised at all levels. The MHA’s experience could be further used to improve healthcare delivery to other minority population groups. Finally, technological complexity is matched by bringing together multiple healthcare providers (hospitals, general practitioners, mental health services, Māori health, and well-being services), who will be encouraged to work together to provide a holistic service for their patients. For example, the health reforms recognise the important place Rongoā Māori — Māori healing based on Matauranga Māori — plays in the NZ healthcare system. Thus, the reforms emphasise the diversity of approaches by which better health and well-being can be achieved.

 In terms of the reform’s objectives, *consistency* is achieved by a higher level of centralisation while *equity* is addressed with the presence of two key independent structures working as equal partners — HNZ and MHA — and unlinking health provision to an individual’s address and geographical location:

 “*People will have access to the right care at the right time regardless of where they live” *(D29).

*People-centred care* and *access to healthcare* are addressed by the formation of localities, which will provide customised and targeted service to their respective populations. This will be achieved through higher autonomy, local population health analytics and partnering with organisations outside the healthcare system to address various causes and contributors to population health such as education providers and NGOs at a local level:

 “*I think the locality concept creates opportunity for people to be focusing on a specific area with a particular lens” *(P7).

 A new PHA is being setup to coordinate nationwide health objectives and outcomes and thus ensuring an overall *coherence* in the system.

####  Weaknesses

 While the proposed structure helps the healthcare system to make a significant step forward in matching complexity of the healthcare, *generative* and *dynamic* complexity are not sufficiently addressed in the reforms. Generative and dynamic complexity indicates how elements outside the boundary of a conventional health system contribute to the overall health and well-being index of a community, thus exposing healthcare to be a non-linear function: many factors that affect population health are outside the MoH’s jurisdiction (eg, housing, employment, and education). The reforms do not sufficiently emphasise coordination with other Ministries and Government departments that deal with such issues which directly affect the health and well-being of the population.

 While official documents and our participants recognise the multiple causes for poor health outcomes, they do not explain how this interconnectedness is going to be structurally addressed:

 “*We need to have all sorts of things that will contribute to people’s health and well-being. Addressing that in a partnership is crucial, but I don’t know if that goes wide enough to date. At this point, nobody knows how far it goes” *(P8).

###  System 2 – Coordination of Activities

####  Strengths 

 The health reforms recognise the complexities of coordination in the existing system and have positioned themselves to eliminate these complexities as one of the primary goals. Participants mentioned that the system has been fraught with a silo mentality and poor integration, which inhibits providing a smooth patient experience and leads to sub-optimal health outcomes. Major coordination mechanisms outlined by the reforms are related to legislation, stronger centralisation, development and use of a nationwide patient management system, creation of a common narrative, and strengthening partnerships and relationships within the system especially partnerships with Māori.

 First, official documents see *centralisation* as a critical coordination and cooperation mechanism among multiple units and entities within the reformed system. Centralisation will also be key in sharing and implementing best practices in the system, thus:

 “*While our response to COVID-19 has been world-leading, it also highlighted weaknesses, particularly that our 12 regional Public Health Units needed better national coordination and leadership when responding to nationwide threats, and to be able to better spread best practice and improvements across the system” *(D46).

 Second, the reforms aspire to introduce a shared patient management system nationwide. Participants saw this as a crucial mechanism to overcome silos in the system and poor communication among different stakeholders and providers. They anticipated improvements in patient handovers and waiting periods with the implementation of this system.

 Third, the development of a common narrative is another coordination mechanism, which has already enabled a shared understanding of the reform’s objectives and has helped to achieve agreement among different stakeholders with a shared ethos within the system.

 Finally, a focus on further strengthening partnership between Māori and non-Māori entities by Māori involvement in the development of future health programmes and strategy is a representation of System 2. The significance of this development at all system levels was highly recognised by official documentation and our participants:

 “*The biggest opportunity that I see for Māori is the partnership, Treaty based nature of the reforms, so we are going to have MHA and iwi partnership boards. That voice, that power given to my people - that is going to be great. It is a huge part of the design. There is the real opportunity there” *(P11).

 Māori involvement will likely improve coordination by addressing cultural tensions and minimising mistrust which has inhibited collaboration in the past.

####  Weaknesses

 While the reforms focus on the issues of coordination and cooperation, the underlying mechanisms that will help to achieve improvements likely need further development.

 Given the level of localisation, assumed autonomy of localities, population diversity and stronger customer voice, centralisation mechanisms may not match the existing complexity and enable smooth coordination within the system as it will require few central authorities to filter vast amounts of information and manage countless information flows.^25^ As a result, participants foresaw tensions between centralisation and localisation predicting negative implications rising from power imbalances:

 “*I think the risk that we are going to get is an imposition of tools that the centre believes will work but won’t work in reality, or be incredibly difficult to implement. I think that can be a real challenge” *(P3).

 Second, the tension between autonomy and centralisation can undermine coordination. A comparison of official documents with interviews highlighted this. Participants saw tensions around responsibility and funding at the locality level, if these details are not properly addressed from the outset. Interestingly, official documents are slow in recognising these tensions. While centralisation was emphasised in the official documents, participants seemed more interested in localisation and autonomy. Such imbalances in priorities indicate the possibility of conflict and the need to develop mechanisms to address it.

 Third, participants mentioned a possibility of transference of the silo mentality, which hinders coordination, from the existing system to the reformed system. Participants stated that they were not aware of any specific approaches within the reforms to address this issue. Moreover, some of the participants pointed to existing competition among health providers which can undermine collaboration. This competition was often associated with the existing funding and mechanisms and contracting. Participants suggested that without a radical change to funding models, partnerships aspired by the reforms cannot be achieved:

 “*But, at the frontline level, it can be difficult, the frontline staff might still not get along and see each other as competition and may see that their jobs depending on not cooperating. So yeah, there are going to be problems. We are trying to work those out but I don’t think we have any organised way around it” *(P9).

 Finally, coordination will be strongly required should dismantling of “an address lottery” bring desirable results. However, official documents do not provide sufficient evidence of these mechanisms to be established except for a nationwide patient system. Participants’ discussion revealed technology alone will not be able to resolve coordination and collaboration problems.

###  System 3 and 3* – Operational Control and Auditing

 To analyse System 3, we analysed resources underpinning achievement of the goals of the reforms (System 3) and the performance management approaches proposed by the reforms (System 3*). Our analysis suggests that this system is the least addressed in the official documents and least understood by the participants (healthcare managers).

####  Strengths 

 In terms of resources official documents primarily discussed investments in *technology*, which included a new patient management system and telehealth services. Participants largely agreed with the need for a patient management system, explaining that the absence of shared databases negatively affects partnerships, collaborations, patient, and health outcomes. Telehealth was not mentioned by our participants as an important resource, though overall technological improvement was advisable.

 While telehealth is emphasised in the official documents as an enabler of health accessibility and equity, it should be noted that telehealth is not fully unpacked in the documents.

*Funding* is deemed another important resource. Official documents provide substantial information about the amount of funding and its division among stakeholders at the central level, indicating that some funding will be also allocated to training and development of digital and other capacities.

 Finally, the reforms discussed *capability development* focusing largely on the leadership and capabilities and technological competences.

####  Weaknesses 

 The main weakness was observed in the lack of a clear implementation plan within the reform design. The implementation part was not clear to the participants either. While some resources were discussed in the reform design, there was a lack of detail and understanding of how they will be provided and what is going to change. Neither funding, nor human resources seen by participants as crucial for the success of the reforms are discussed in official documents in detail. Participants expressed concern that the funding system might not change, preserving competition and keeping patients with high needs from accessing primary care. Even with a focus on supporting Māori health *who* will be responsible for service delivery and *how* it will be funded was not clear:

 “*What are we going to do moving forward if we’re right now being funded to provide services for Māori through that services to improve access budget, then, if we no longer have that part of the budget, then, how do we or who’s going to service that population? How will it be done? And will it be like all Māori services can support all Māori? So am I supposed to say sorry I can’t work with you?” *(P8).

 In terms of human resources participants advocated for the increase of workforce and recruitment of new people. Health workforce shortage is well documented in NZ and is seen to contribute to inefficiencies in the existing system.^67^ Indeed, we observed this problem is largely discussed by news articles highlighting nursing, midwifery and medical shortages,^68-74^ closures and reduced hours of hospital wards,^75-78^ and providing individual stories of overworked and stressed workforce.^68,79,80^ Interviewees also clearly mentioned a shortage of human resources and a need to increase the trained health workforce. It was also argued that new employees are needed to ensure that a new culture and mindset are established in the reformed system:

 “*We need new blood too. I would hate to see the new system with all the old organisations, old people from DHBs or whatever. That is not new to me. So yeah that will hinder [progress] too. We need new mind-set” *(P4).

 Interestingly, official documents were not very clear about addressing these workforce shortages. They mostly discussed the transfer of existing staff to the new system. In the same vein, capability development was mostly focused on provision of training for the existing workforce. This is an indication that the workforce-related concerns are overlooked in the reforms, which may result in the reformed system being underprepared to meet the ever-changing environmental conditions and challenges including the growth and aging of the NZ population.

###  System 3* – Auditing

####  Weaknesses 

 Our analysis indicated that there was a lack of information regarding performance measures that may be used in the system to analyse its achievement of the objectives and ability to follow a strategic plan. For example, while equity is a central theme and objective of the reforms, how it will be analysed or measured is completely missing. The only indicator of inequity discussed in the official documentation is the average life-span of Māori, Pacific peoples, and non-Māori communities. Since health and well-being is a complex phenomenon characterised by multiple contributing factors and non-linearity, an immediate change in this indicator due to the health reforms is unlikely. Interim measures are required but not identified.

 Similarly, patient-centred care and patient experience are highlighted as primary objectives too, yet no information about how to measure these was available: It might be suggested that customer feedback is going to be used as one of the monitoring mechanisms, however there is no evidence of how capturing such feedback will serve as a performance measure.

 “*I don’t think they have included anything on evaluation, but it’s a really good point. When I had our conversations with […] it didn’t come up. I don’t remember seeing anything about evaluation so far” *(P6).

 Moreover, a lack of reference to any performance measures which informed the reforms’ development hint that the performance measurement is not fully incorporated in the current system either. Whether it is a capability to be developed by the reforms is not clear.

###  System 4 – Development 

####  Strengths 

 Several mechanisms are outlined in the reform documents to ensure further development. These are: a commitment to invest in system intelligence, continuing innovations prompted by COVID-19, greater involvement of Māori in the health system design and provision, dissemination of best practice, and listening to consumer voice.

 First, the official documents claim commitment to learning. Learning and especially double-loop (questioning assumptions behind the systems and changing these assumptions and values which result in the change of actions^81^) learning is an important function of System 4. A recurring theme in the documents was that the NZ health system has learned a great deal from how it responded to the COVID-19 outbreak. This included finding innovative ways to continue health delivery and managing the burden on health services. This learning informed the development of the reforms to improve the adaptability of the system for future needs. Also, experimentation and pilot projects are a part of the reforms.

 Second, there is an intention to harness the best practices which exist in the system. It was suggested that the reforms will ensure that the best practice is easily disseminated among various entities primarily due to increased centralisation.

 Third, the establishment of MHA will create a unique opportunity to learn how to engage and improve the health outcomes of diverse population groups in NZ:

 “*I should add that we need equity too which is around working with Māori, and you know I think if we get that right, it will flow on to other minorities” *(P1).

 Fourth, consumer voice could be seen as an important enabler of health system development. Constant feedback from consumers of the health system should allow better understanding and agile response to the changing consumer needs.

####  Weaknesses 

 While development is supported by the reform’s design its success depends on the smooth functioning of other systems and our participants indicated some potential caveats in System 4. Given the existing challenges with collaboration and data sharing, development and change might require a long time to initiate.

 Some participants believed that the new system will not procure double-loop learning due to the lack of a systemic approach to development:

 “*I know that they actually want to take something that is either working or has the potential to work. So I don’t believe we’re looking at completely reinventing the wheel here. With this government, this is about seeing whether it sticks or not” *(P14).

 This aligns with the participants’ concern that the reforms are not emphasising enough the need for a fresh mind-set. Also, some participants worried that the positive aspects of the current system are not going to be incorporated in the reforms. Finally, a healthcare workforce shortage can significantly impede development with people focusing on current activities rather than developing future strategies.

###  System 5 – Governance

####  Strengths 

 Within System 5, there is a high level of communication of system goals and objectives, and a strong consensus on these goals is present. These goals are re-iterated in the official documents and by our participants.

 Another strength of the governance system is related to the variety of approaches to ensure improvements in health delivery. Among these approaches are high level of centralisation, use of expert opinion, development of partnerships and active engagement with communities especially with Māori and Pacific peoples.

####  Weaknesses 

 While communication regarding the reforms objectives has been extensive — provided in both Te Reo (Māori) and English — the communication about the implementation of the reforms has fallen behind. Official documents do not discuss implementation plans in detail and the participants did not have any details either. While there is a three-phase plan for the transition, there is no discussion about how the reforms are going to function to achieve their objectives. And while the only concrete implementation plan available in the documents was about the localities; participants recruited from potential localities felt a high level of frustration due to a lack of communication:

 “*So I think most of the information that we got from them was very much on how the system will look like but it didn’t inform us how the system will operate. That’s the crucial difference” *(P11).

 Participants admitted that they mostly received information about the reforms through informal channels instead of the government’s transition unit. As a result, there was a lack of preparation and where the preparation was happening there was no assurance that it was aligned with the government vision. Our overall findings are summarised in Table 3.

**Table 3:** Summary of Findings Utilising the Viable System Model Framework

**VSM Systems**	**System in Focus: Health Reforms**	
	**Sub-System Strengths**	**Sub-System Weaknesses**
System 1	Geographical and customer complexity addressed Focus on consistency, equity, people-centred care, and accessibility	Generative and dynamic complexity not fully addressed Lack of acknowledgement of various factors (outside the health system) contributing to health and well-being of NZ populations
System 2	Legislation to support the reforms Centralisation Patient information system Strengthening of partnerships HNZ, MHA, and Māori Iwi boards Development of the common narrative	Tension between centralisation and autonomy – imbalance in priorities Silo mentality Funding mechanisms which contribute to competition Lack of clarity about coordination after the removal of “address lottery”
System 3 and 3*	Investment in technology Funding mechanisms Capability development	No explicit implementation plan provided Lack of detail about funding mechanisms Low focus on healthcare workforce shortage problem Lack of performance management and measurement system
System 4	Commitment to invest in system intelligence Learning from COVID-19 response Greater involvement of Māori with the potential to spread learning to work with other groups Dissemination of best practice Listening to consumer voice	Lack of focus on a fresh mind-set Lack of systemic approach to development Problems is other related systems (eg, shortage of healthcare staff, silo-mentality, competition)
System 5	Communication about problems facing the current system and objectives of the reforms High level of consensus Shared identity Variety of approaches Hierarchy—centralisation and experts involvement Egalitarianism—customer forums and feedback, provider engagement, and codesign Individualism—locality prototypes, tendered grants, and contracts	Lack of communication about the implementation of reforms Uncertainty about delivery and responsibilities

Abbreviations: VSM, viable system model; NZ, New Zealand; HNZ, Health New Zealand; MHA, Māori Health Authority.

## Discussion

 This study evaluates the 2022 NZ *Pae Ora* health reforms using the VSM as an analytical framework and makes several contributions to the literature. First, it systematically evaluates the health reforms at an early stage allowing the identification of the health system’s design features which contribute to its viability. The use of the VSM helped us to understand how well the reforms address the intricacy of public health and well-being, and the complexity of the healthcare environment. Shortcomings of the proposed reforms were also recognised. If these shortcomings are not addressed they can undermine the objectives of the reforms preventing delivery of equitable and better healthcare outcomes with associated weakening public trust and change fatigue.^82-84^ Second, our study uses the VSM in a somewhat novel context of national health reforms. VSM has not been widely used in healthcare,^28^ and is often applied at an organisational level.^22^ Consequently, our study demonstrates the applicability of VSM in a healthcare setting at a national level. Finally, by structuring the decision problem,^25^ this study can initiate a much needed conversation among multiple stakeholders on how to ensure the reforms achieve their ambitious goals and stay viable.

 Although an earlier evaluation of the previous health system funded by the government, known as “The Simpson Report,” along with many experts, suggested to reduce the number of DHBs to reduce the structural complexity,^85^ none of them anticipated or proposed a radical change prescribed in the NZ *Pae Ora* reforms. Our analysis suggests that this radical change, while offering important benefits, created some challenges which were not well addressed in the design. We have identified significant imbalances in the development of systems 1 to 5 of a new Health system. We find that while the reforms’ design makes systems 1 and 5 particularly strong, systems 2 and 3 are fraught with challenges.

 With respect to system 1, the design of the NZ health reforms appreciates the complexity of the healthcare environment — multiple stakeholders, social inequalities, interdependencies, etc — as well as the “wickedness”/complexity of healthcare problems.^1,16,17,29,41^ This complexity is addressed by the new structure of the reformed system and the development of the relevant governance institutions. Here the development of localities is of particular strength as they aim to address geographical complexity and ensure that health and well-being needs and interests of diverse NZ populations are met. At the same time, establishment of MHA specifically focuses on the needs of Māori. Finally, Ministry of Disabled People gives voice to this group of consumers.

 System 5 also seems to be well designed. The findings suggest that it well communicates goals and objectives of a new system ensuring that they are shared within the system. The literature suggests that governance structures which combine the features of hierarchy, fatalism, egalitarianism, and individualism are particularly well positioned to address complex problems.^19,41^ The reforms demonstrate an attempt to develop governance system with at least three of these features. Hierarchy is captured by the presence of centralised agencies (HNZ, PHA, and MHA) and high involvement of experts. For example, the NZ government recruited consultants and experts to inform and implement the transition to the new reformed system.^86,87^ Egalitarianism is represented by HNZ and MHA having equal rights and voice, engagement with communities and providers through customer forums, feedback channels, and stronger ties between localities and communities. Individualism is represented by the heightened levels of autonomy and elements of competition among providers preserved by old funding mechanisms.

 However, while systems 1 and 5 are well developed within the reforms’ design, much less attention was devoted to the development of systems 2 and 3. Indeed, the official documentation, which could be seen as a strategy for NZ healthcare sector, lacks details regarding the implementation and operationalisation of reforms—another important aspect of a strategy.^88^ Thus, systems 2 and 3, which are more focused on operationalisation and day-to-day activities of a system, reveal significant weaknesses. The VSM posits that for a system to be viable, all its five sub-systems need to be strong,^26^ but our analysis suggests that NZ health reforms, despite their strengths, do not satisfy this crucial requirement. Moreover, significant imbalances in the strength of sub-systems may undermine the system’s viability even more than a weak development of all subsystems.^89^

 The weaknesses of systems 2 and 3 identified by our analysis suggest that if not addressed they might infiltrate the reformed system with the persistent problems of healthcare.^44,90-92^ For example, with respect to system 2 current coordination-related problems such as silo mentality and competitive funding approaches might persist.^28,93,94^ However, achievement of equity goals will require a strong collaboration.^50^ At the same time the reformed system might create additional coordination problems by simultaneous increase in centralisation and local autonomy.^22^ In addition, the removal of “address or postcode lottery” will require further coordination mechanisms within the health system. However, any evidence of the need for such mechanisms being recognised and incorporated into the reform’s design was not found.

 Furthermore, the official documentation does not sufficiently discuss how the reformed system will be resourced (System 3) and resourcing problems of the previous system will be addressed. Human resources are a major concern in the NZ health system. There are severe workforce shortages across primary and secondary care.^95-99^ However, these are not addressed by the reforms. Even when such issues are mentioned in the reforms, no mechanisms or strategies to resolve them are discussed. These problems spill over to system 4 which does not recognise how the resourcing problem will be addressed to enable future development of the health system. This puts the long-term sustainability of the health system in jeopardy^100^ with some predicting system’s overstretch.^50^

 Finally, performance measurement (System 3*) is overlooked almost completely. This is not wholly surprising, as health sectors tend to lack comprehensive performance management systems.^101-103^ However, measurement becomes even more important when any change is introduced as they enable to evaluate its effectiveness.^28,104^ In the case of these health reforms how the achievement of the reforms’ objectives will be monitored is practically absent. For example, performance metrics to monitor the effectiveness of new agencies and localities are lacking. While official documents discuss increased funding as a proxy measure of the success of reforms, especially when it comes to Māori and Pacific peoples’ health, funding does not constitute a measure of performance in itself.^105^ The second measure highlighted here is life-expectancy. While this is an important health outcome, social determinants outside the boundary of a health system contribute to this measure—a fact acknowledged in the official documents and our participants.^106-108^ It will be problematic to delineate the impact of health reforms on this metric. This generative complexity is similarly overlooked by System 1. The identified weaknesses highlight significant challenges in reforms’ implementation and call for further elaboration of reforms.^50^

 This study has several limitations. First, the recruitment of participants was a challenging task. While we did not set a particular number of participants for the study, we were able to recruit only a small number because the majority of potential participants declined our invitation explaining that they did not have any reliable information to share. However, some of the approached participants who declined our invitation recommended to contact their colleagues who may have more information. This challenge reiterates one of the system weaknesses highlighted by our analysis – poor communication of the change. Second, in this study we interviewed only representatives of PHO management, therefore perspectives of other PHOs (eg, hospitals, mental health support services), non-managerial staff, as well as the customers of the healthcare system were not incorporated. Also, the government perspective was largely represented by the official communication. Third, we were able to analyse only publicly available documents on the reforms. While they contained detailed information, there can be some documents not publicly available. Some of them may be addressing concerns raised in this paper. However, this seems unlikely as our health sector participants — direct stakeholders of the reforms — were not aware of such documents. Finally, VSM does not specifically focus on the role of culture in systems’ viability, while culture might be especially important in the design and implementation of change in healthcare.^109^ In case of our study it would be useful perhaps to look at the national culture and its role in the viability of health reforms’ design. While, we have not done so specifically, based on our findings, it might be suggested that design of VSM’s sub-systems reflects the cultural aspects. For example, the principles of egalitarianism – one of the features of NZ culture were strongly underpinning sub-system 1, 2 and 5 as well as influencing the design of communication channels between the health system and the environment (eg, increased consumer involvement).^110^ Relatedly, the values of biculturalism which the reforms sought to promote were reflected in the organisation of sub-system 5.^111^ We also suggest that the aforementioned principles and values strengthened the relevant sub-systems. Future studies might specifically focus on the cultural aspects of health reform design and their impact on the reforms success and viability, given that some researchers suggest that system’s viability is closely linked to culture.^89^

###  Implications for Policy

 The findings of this research, especially those that demonstrate the shortcomings of the reforms design can be used by policy makers to advance the reforms. In particular, our findings suggest that special consideration should be given to resourcing, performance management and measurement if equitable health outcomes for Māori and Pacific peoples are to be achieved. Funding is touted as a key driver of the health reforms, especially for NZ’s marginalised populations. While increased funding should help in theory to deliver equitable outcomes this may be undermined by the absence of measures or metrics to analyse the efficacy of increased funding. Without that, increased funding will not necessarily result in better outcomes. In fact, it can hide operational and systems-level inefficiencies and inadequacies that further diminish the performance of a system. It is advised that if such challenges are not addressed, theimplementation of the *Pae Ora* health reforms will be seriously jeopardised.

 Furthermore, due to the reforms’ intention to increase centralisation while affording more autonomy, certain tensions between the structures and the two ideologies (centralization vs autonomy) may arise.^22^ The VSM can be instructive in addressing these tensions. Since viable systems have recursive structure,^26^ ensuring that polices, practices, standards and missions are developed and discussed at all levels and that they match each other can help to address the centralisation-autonomy tension. Also, VSM suggests that development of communication channels between the larger system and embedded systems is of importance. There is a need to ensure that this communication channel is not overloaded and that mechanisms related to systems 2 and 3* (coordination, sporadic auditing, and performance measures) are also engaged to help preserve autonomy while ensuring cohesion within the healthcare system.^112^ Finally development of various communication channels with the entities outside healthcare system (environment) can help to address better the generative complexity. These communication channels need to be developed at all levels of the health system (health organisation, localities, HNZ, MHA, and MoH). This match, and link, between the external and internal communication channels can help to improve agility in an everchanging environment.^113^

## Conclusion

 This study analyses the 2022 NZ *Pae Ora* health reforms using the VSM as an analytical framework to recognise the strengths and weaknesses of various aspects of the reforms’ design. The intent was to generate useful information that may aid the government and the new national providers to improve the reforms’ delivery at the earliest stages. We conclude that the health system reforms may not lead to a viable NZ health system going forward.

## Acknowledgements

 The authors would like to thank all the participants who provided their valuable time and insights in this research.

 The author also thank the Professor Ali-Akbar Haghdoost and Sahar Najafizadeh of IJHPM for providing support throughout the process. We also thank the five reviewers who provided highly valuable feedback that helped us to improve this paper significantly. Finally, the authors thank Moira, Dale, Liz, Elise, and Stephanie. This is our wonderful admin team. Nothing would happen without them.

## Ethical issues

 Ethical approval was obtained from the University of Otago Human Ethics Committee (D21/271). Written informed consent was obtained from all participants.

## Competing interests

 Authors declare that they have no competing interests.

## Funding

 This study was funded by the Health Research Council of New Zealand (HRC Project Grant 20/875). The funding body has not had any role in design of the study or outputs from the study.

## Supplementary files


Supplementary file 1. Details About Analysed Secondary Data.

Supplementary file 2. Interview Schedule.

Supplementary file 3. Pre-reforms Health System.
