# Supplementary material for: New Zealand Pae Ora Healthcare Reforms 2022: Viable by Design? A Qualitative Study Using the Viable System Model
Source: Int J Health Policy Manag. 2023 Dec 6;12:7906. doi: 10.34172/ijhpm.2023.7906 (PMC10843487; doi:10.34172/ijhpm.2023.7906)
Supplement: Supplementary file 1 — Details About Analysed Secondary Data. [file ijhpm-12-7906-s001.pdf]

Article title: New Zealand *Pae Ora* Healthcare Reforms 2022: Viable by Design? A Qualitative Study Using the Viable System Model

Journal name: International Journal of Health Policy and Management (IJHPM)

Authors' information: Adeel Akmal[1,2]*, Nataliya Podgorodnichenko[3], Robin Gauld[4], Tim Stokes[1]*

[1]Department of General Practice and Rural Health, Dunedin School of Medicine, University of Otago, Dunedin, New Zealand.

[2]Department of Business Studies, University of Iceland, Reykjavik, Iceland.

[3]DBA Programme, Otago Business School, University of Otago, Dunedin, New Zealand.

[4]Department of Management, Otago Business School, University of Otago, Dunedin, New Zealand.

***Correspondence to:** Adeel Akmal; Email: adeel.akmal@otago.ac.nz & Tim Stokes; Email: Tim.stokes@otago.ac.nz

Citation: Akmal A, Podgorodnichenko N, Gauld R, Stokes T. New Zealand *Pae Ora* healthcare reforms 2022: viable by design? a qualitative study using the viable system model. Int J Health Policy Manag. 2023;12:7906. doi:10.34172/ijhpm.2023.7906

Supplementary file 1. Details About Analysed Secondary Data

The news articles were collected using Factiva® database (Date: 11 April, 2022) with the following search string:

"Health New Zealand" OR "Maori Health Authority" OR "Māori Health Authority"

Type of documents	Number of documents	Source(s)
Government documents	99	futureofhealth.nz
Newspapers	237	New Zealand Herald – 82<br>NZ Doctor – 39<br>Dominion Post – 58<br>RNZ – 31<br>Otago Daily Times – 27
