# Supplementary material for: New Zealand Pae Ora Healthcare Reforms 2022: Viable by Design? A Qualitative Study Using the Viable System Model
Source: Int J Health Policy Manag. 2023 Dec 6;12:7906. doi: 10.34172/ijhpm.2023.7906 (PMC10843487; doi:10.34172/ijhpm.2023.7906)
Supplement: Supplementary file 2 — Interview Schedule. [file ijhpm-12-7906-s002.pdf]

Article title: New Zealand *Pae Ora* Healthcare Reforms 2022: Viable by Design? A Qualitative Study Using the Viable System Model

Journal name: International Journal of Health Policy and Management (IJHPM)

Authors' information: Adeel Akmal[1,2]*, Nataliya Podgorodnichenko[3], Robin Gauld[4], Tim Stokes[1]*

[1]Department of General Practice and Rural Health, Dunedin School of Medicine, University of Otago, Dunedin, New Zealand.

[2]Department of Business Studies, University of Iceland, Reykjavik, Iceland.

[3]DBA Programme, Otago Business School, University of Otago, Dunedin, New Zealand.

[4]Department of Management, Otago Business School, University of Otago, Dunedin, New Zealand.

***Correspondence to:** Adeel Akmal; Email: adeel.akmal@otago.ac.nz & Tim Stokes; Email: Tim.stokes@otago.ac.nz

Citation: Akmal A, Podgorodnichenko N, Gauld R, Stokes T. New Zealand *Pae Ora* healthcare reforms 2022: viable by design? a qualitative study using the viable system model. Int J Health Policy Manag. 2023;12:7906. doi:10.34172/ijhpm.2023.7906

Supplementary file 2. Interview Schedule

Organisation and Role

Could you start by telling me about your role in the XXXX organisation?

How long have you been in the role?

How are you, in this role, involved in the locality partnerships development and the transition to the new health new Zealand system?

Aims and Objectives of Locality partnerships

From your perspective, what do you see as being the key aims and objectives of locality partnership working?

    What do you see as being the key reasons behind developing locality partnerships?

How do you think locality partnerships and the new system differs from DHB and alliance networks (the old system)?

    How are they structured?

What do you see as being the value of locality partnerships? Or Perhaps, what benefits do you see coming out of this arrangement?

What do you see as the role of MOH and other governmental organisations in contributing towards locality partnerships?

    How much information, help and direction are you getting from them?

    Would you like more input and assistance from them?

Are you in communication with any other PHO or DHB who is going through the same changes and sharing notes?

Operational Working of Locality Partnerships

Something we're interested in understanding is *how* locality partnerships will actually work? Their administration and management within a bigger and broader system and how they will interact with neighbouring localities. How will they bring about improved system and health outcomes? Could you please tell me about how you see them operating on their own as well as part of the broader health system?

How will that bring about better health outcomes for the populations?

What local organisations and providers are you working with to form locality partnerhsips?

Could you tell me about how XXXX will go about achieving the objectives you described?

Are you conducting any pilot studies to ensure these results and to tweak out other organisational and operational issues?

Could you tell me about the approaches and processes locality partnerhsips will use to promote improvements in health outcomes, integration and equity?

Do the approaches you use vary depending on whether you are working on long or short term objectives? If so, how?

What are the short-term and long-term goals at the moment in your district?

How do you decide what areas of work to prioritise? Who is involved?

Are there any performance measures that you are developing or perhaps coming from MOH to inform operational excellence in locality partnerhsips working? Any examples?

Enablers and Challenges

What do you consider will enable progress towards locality partnerhsips working?

In your region/nationally?

How are you promoting or supporting these factors within your organisation?

What do you consider to be the major challenges or barriers to locality partnerships being fully operationalised?

Do you have any strategies in place to ensure you are ready to overcome them?

Is there anything about the organisational structure, culture and leadership of the XXXX that you think will either supports or hinders locality partnership working?

Anything unique in your organisations that will make things work?

Do you find there are any factors that either help or hinder your ability to carry out your locality partnership formation and development? (e.g. time constraints, support of colleagues)

How you think Covid19 has impacted this transformation?

What do you consider to be the most significant impact?

Has Covid19 highlighted any particular challenges or strengths with respect to the locality partnership development in your region? In what way?

How have you worked through these challenges? Who led the response?

How did you decide what to prioritise? (if relevant)

Outcomes

What outcomes are you anticipating out of the locality partnerships?

Could you tell me how you think locality partnerships will make a difference to health care integration, outcomes or health care equity?

Are there any other areas where you think locality partnerships working is making a difference?

How will you go about monitoring progress toward improved locality population outcomes?

Are there specific or standardised outcome measures you use to monitor progress?

Do you have a view on how useful these measures are?

Do the measures you use vary for long- or short-term outcomes?

Are there any areas of work that you consider to be valuable but currently are not measured?

How would you measure the value of locality partnerships?

Is there anything else you would like to comment on in relation to DHB regional working in general or locality partnership?
